# Iron (Fe)-doped mesoporous 45S5 bioactive glasses: Implications for cancer therapy

**DOI:** 10.1016/j.tranon.2022.101397

**Published:** 2022-03-30

**Authors:** Farzad Kermani, Arghavan Vojdani-Saghir, Sahar Mollazadeh Beidokhti, Simin Nazarnezhad, Zahra Mollaei, Sepideh Hamzehlou, Ahmed El-Fiqi, Francesco Baino, Saeid Kargozar

**Affiliations:** a Department of Materials Engineering, Faculty of Engineering, Ferdowsi University of Mashhad (FUM), Azadi Sq., Mashhad 917794-8564, Iran; b Tissue Engineering Research Group (TERG), Department of Anatomy and Cell Biology, School of Medicine, Mashhad University of Medical Sciences, Mashhad 917794-8564, Iran; c Genetics Laboratory, Ghaem Hospital, Mashhad University of Medical Sciences, Mashhad, Iran; d Hematology/Oncology and Stem Cell Transplantation Research Center, Tehran University of Medical Sciences, Tehran, Iran; e Glass Research Department, National Research Centre, Cairo 12622, Egypt; f Institute of Materials Physics and Engineering, Applied Science and Technology Department, Politecnico di Torino, Corso Duca degli Abruzzi 24, 10129 Torino, Italy

**Keywords:** 45S5 bioglass, Mesoporous bioactive glasses (MBGs), Cancer treatment, Fenton's reaction, Bone tissue engineering

## Abstract

The utilization of bioactive glasses (BGs) in cancer therapy has recently become quite promising; herein, a series of Fe-doped mesoporous 45S5-based BGs (MBGs) were synthesized via the sol-gel method in the presence of Pluronic P123 as a soft template. The physico-chemical and biological properties of the prepared glasses were well-characterized through structural assessments, thermal analyses, and electron microscopic studies. Electrochemical analyses, including cyclic voltammetry (CV) and electrochemical impedance spectroscopy (EIS), were also performed to investigate the actual potential of the Fe_2_O_3_-containing MBGs in modulating the Fenton's reaction. The XRD results confirmed the glassy state of the Fe-doped samples before immersion in simulated body fluid (SBF). The prepared Fe-doped MBGs exhibited a particle size in the range of 11–86 nm, surface charge of 27–30 mV, S_BET_ of 95–306 m^2^/g, and M_s_ of 0.08 to 0.2 emu/g. The incorporation of Fe_2_O_3_ led to a negligible decrease in the bioactivity of the glasses. The CV analysis indicated that the Fe-doped MBGs could generate H_2_O_2_ in a cathodic potential higher than -0.2 V (vs. Ag/AgCl) in the O_2_-saturated Na_2_SO_4_ solution. Additionally, the data of the EIS test revealed that the Fe_2_O_3_-doped MBGs could increase the standard rate constant of Electro-Fenton's (EF) reaction up to 38.44 times as compared with the Fe-free glasses. In conclusion, Fe-doped 45S5-derived glasses may be useful in cancer therapy strategies due to their capability of activating Fenton's reaction and subsequent production of reactive oxygen species (ROS) such as ^•^OH free radicals.

## Highlights

• Fe-doped mesoporous 45S5 BGs were successfully synthesized using the sol-gel route.
• Fe-doped MBGs exhibited a particles size of 12 nm with a high surface area of 306 m^2^/g.
• Fe-doped MBGs could generate H_2_O_2_ in a cathodic potential higher than −0.2 V.
• Fe-doped MBGs increased the standard rate constant of Electro-Fenton's (EF) reaction up to 38.44 times as compared with the Fe-free glasses.

## Graphical abstract

**Figure:**
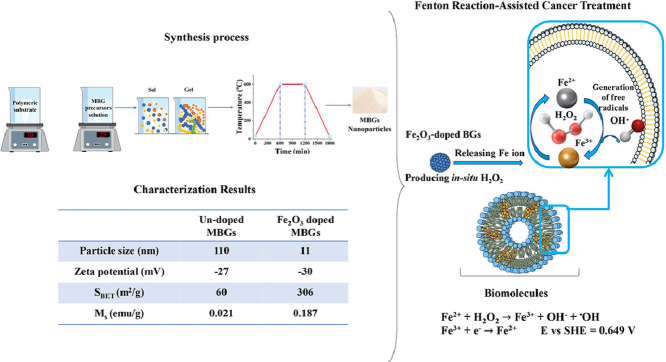

## Introduction

Cancer is still the second leading cause of life-threatening human morbidity over the world, with an estimated number of nearly 10 million deaths in 2020 [1], [2], [3]. Among different types of cancer, bone tumors (e.g., osteosarcoma) form approximately 5% of childhood and 1% of adults’ cancers [4]. Up to date, a huge number of attempts have been made to treat bone cancers, including chemotherapy, radiotherapy, and surgical excision. The mentioned therapeutic strategies have led to the enhanced survival rate of patients; however, novel therapies are required to simultaneously battle against cancer cells and restore the lost bone tissue [5]. On this matter, designing and developing anticancer biomaterials, under the concept of tissue engineering, hold great promise in the biomedical setting [5,6].

As biocompatible materials, bioactive glasses (BGs) have been extensively applied for managing various bone lesions over the past 50 years. The first developed member of the BG family was a silicate-based glass, the so-called 45S5 (Bioglass®), with the composition of 45% SiO_2_, 24.5% Na_2_O, 24.5% CaO, and 6% P_2_O_5_ in weight% [7,8]. There is a long history of successful usage of 45S5 BG in the medical setting; other types and formulations of BGs have been developed over the years for different applications [9]. In fact, the reason for such achievement is associated with the excellent characteristics of BGs for hard tissue engineering, including their osteoconductive and osteoinductive properties. More importantly, BGs show a unique feature for regenerative medicine, i.e., their capability of bonding to human tissues, known as “bioactivity.” Over time, specific formulations of biocompatible glasses were developed to further expand and tune their biological properties for specific conditions in the body, such as cancer treatment [10], [11], [12]. In this regard, a series of metallic and non-metallic elements can be added to the basic composition of BGs to render anticancer activities [13], [14], [15]. For instance, iron (Fe) was successfully incorporated in glasses for rendering osteostimulatory multi-functions, such as improved bone metabolism through enhanced calcification, osteoblast proliferation, as well as apatite-forming ability [16], [17], [18]. Notably, Fe also shows significant anticancer activity in its oxide compounds (e.g., Fe_2_O_3_). In fact, this element may trigger specific chemical reactions (e.g., the Fenton's reaction) which can eventually lead to tumor cell death.

ROS have widely been studied and utilized in cancer therapy; they comprise both oxygen-related free radicals (e.g., hydroxyl radical (^•^OH)) and non-radical species (e.g., singlet oxygen (^1^O_2_)) [19]. Although high amounts of ROS play roles in up-regulating pro-tumorigenic signals and improving tumor proliferation, they can also activate anti-tumorigenic signaling pathways to increase cancer cell death [20]. It has been demonstrated that Fe ions (Fe^2+^/Fe^3+^) under a redox mechanism can increase cancer cell death due to their ability to generate ROS, which is based on transforming less toxic H_2_O_2_ to cytotoxic ^•^OH [16,21]. Therefore, there has been growing interest in utilizing Fe_2_O_3_-containing formulations in cancer therapy approaches [21] and, for example, adding Fe oxides to biocompatible glasses and glass-ceramics has become a highly-promising procedure in such kind of biomedical engineering settings [22].

In the present study, we, for the first time, synthesized a series of Fe-doped 45S5-based mesoporous BGs (MBGs) for potential applications in cancer therapy based on triggering Fenton's reactions. For this aim, we utilized the sol-gel method for producing silicate-based MBGs in which Fe_2_O_3_ has been added in concentrations of 0, 1, 2.5, 5, and 7.5 mol%. The effects of Fe doping on the physicochemical properties of the glasses were studied through a complete series of tests (e.g., DTA-TG, XRD, FTIR, PSA, Zeta potential, FE-SEM, AFM, and VSM). More importantly, our study highlights the effect of Fe-doped MBGs on the properties and the rate of electro-Fenton's (EF) reaction using a series of electrochemical analyses involving cyclic voltammetry (CV) and electrochemical impedance spectroscopy (EIS).

## Experimental

### Synthesis of Fe-doped glasses

The Fe-doped 45S5-based MBGs were synthesized in a 46.14SiO_2_-(26.91-X) CaO-XFe_2_O_3_–54.4Na_2_O-2.55P_2_O_5_ (*X* = 0, 1, 2.5, 5, 7.5 mol%) multi-component system (Table 1). For this purpose, appropriate amounts of reagents, including tetraethyl orthosilicate (Si(OC_2_H_5_)_4_, TEOS), Ca(NO_3_)_2_^•^4H_2_O, Fe(NO_3_)_3_^•^9H_2_O, NaNO_3_, triethyl phosphate ((C_2_H_5_)_3_PO_4_, TEP) were defined using HSC chemistry® software (HSC chemistry® 9.4, Outotec, Espo, Finland) to obtain 10 g of each MBG. The reagents were purchased as an analytical grade substances (Sigma-Aldrich, USA). Experimentally, 2 g of P123 (EO20-PO70-EO20, M_W_ = 5800 g/mol) (Sigma-Aldrich, USA) was dissolved in 77 mL of absolute ethanol (Merck, Germany) in an acidic condition (0.5 mL of HCL (1 M)), named solution 1. Then TEOS and TEP were hydrolyzed in the presence of deionized water and HNO_3_ for 60 min and separately added to solution 1. After that, the nitrate precursors were dissolved in deionized water and added to solution 1 every 45 min intervals. In the next step, the pH of solution 1 was increased up to 14 by adding ammonium hydroxide (25% NH_3_ in H_2_O) in order to immediately form the gels. The formed gels were stirred for 24 h and then aged in sealed bottles for 7 days at room temperature. Subsequently, the mentioned gels were rinsed 5 times with distilled water to remove residual ammonia solution and ethanol. The samples were then dried using the freeze-drying process for 48 h. Next to this step, the samples were initially treated at 250 °C for 24 h and, finally, heat-treated at 600 °C (heating rate of 1 °C/min) in the air for 30 h.

**Table 1:** Nominal compositions in mole % of the undoped and Fe-doped 45S5 MBGs.

Sample code	SiO_2_	CaO	Fe_2_O_3_	Na_2_O	P_2_O_5_
Fe0	46.14	26.91	0	24.4	2.55
Fe1	46.14	25.91	1	24.4	2.55
Fe2.5	46.14	24.41	2.5	24.4	2.55
Fe5	46.14	21.91	5	24.4	2.55
Fe7.5	46.14	19.41	7.5	24.4	2.55

### Characterizations

#### Thermal behavior

The thermal behavior of the freeze-dried samples was analyzed using thermogravimetric (TGA) and differential thermal (DTA) analyses (STA 503, Germany) with a heating rate of 10 °C/min in air. The glass transition temperatures (Tg) of Fe-containing silicate-based BGs were then calculated and compared with those reported in SciGlass database version 7.12 [23]. It is noted that we removed the dropped data (maximum 10%) in order to increase the analysis accuracy, and the resulting values were processed using SPSS Modeler (version 18.22, IBM, USA).

#### XRD analyses

The structure of the synthesized glass particles before and after immersion in simulated body fluid (SBF; see also section 2.3.1) was investigated using X-ray diffraction (XRD) analysis (D8-Advance Bruker, Germany). The instrument conditions were set as 2θ range of 20–80° with Cu-Kα radiation, a step size of 0.05°, and time per step of 2 s. The phase characteristics were studied using the Rietveld refinement method using Profex and BGMN packages [24].

The rate constant (K) of the phase transformation of the Fe-doped MBGs to hydroxyapatite during immersion in SBF was calculated using Equation 0.1 (Eq. (1)) [25].

(1) K=−ln(Ca/C0)/T

where C0 is the initial amount of the hydroxyapatite after the first incubation time (24 h or 86,400 s) and Ca is the amount of the crystalline phase after a time T.

#### FTIR study

The chemical bonding in the structure of Fe-doped MBGs before and after immersion in SBF was investigated using Fourier-transform infrared spectroscopy (FTIR) (Thermo Nicolet AVATAR 370, USA) over the range of 400–4000 cm^−1^.

#### Particle size and zeta potential measurements

The glass particle size was determined by using dynamic light scattering (DLS) (Vasco3, Cordouan, France) analysis. The zeta (ζ) potential of the samples was measured using Zeta potential analyzer (NANO-flex® II, Thermo Fisher Scientific, USA). To do this, 0.01 g of the Fe-doped MBGs were dispersed in 10 mL of absolute ethanol with the aid of ultrasonic waves (FR USC 22 LQ, 400 w, 20%, Taiwan) for 5 min.

#### Morphological observations by electron microscopy

The surface morphology of gold-sputtered MBGs was observed using field-emission scanning electron microscopy (FESEM) analysis (MIRA3, TESCAN, CZ) before and after immersion in SBF. The effect of adding Fe_2_O_3_ on the particle size and morphology was also assessed using transmission electron microscopy (TEM) (EM 208S, Philips, Netherland). The influence of Fe dopant concentrations on the surface topography and roughness was investigated by using atomic force microscopy (AFM) analysis (Nano Wizard II; JPK Instruments, Germany). In order to perform TEM and AFM analyses, 0.01 g of the glasses were first dispersed in 30 mL of absolute ethanol under the action of ultrasonic waves (FR USC 22 LQ, 400 w, 20%, Taiwan) for 10 min. Then, a drop of the prepared suspension was coated on the standard carbon-coated copper meshes grade for TEM and AFM analyses.

#### N_2_ adsorption-desorption analysis

The textural properties of the glasses, including specific surface area (S_BET_), pore size ranges, and pore volume, were determined by nitrogen adsorption-desorption measurement using the Brunauer-Emmett-Teller (BET) and Barrett-Joyner-Halenda (BJH) analysis (Quantachrome instrument, Japan). Prior to performing the analysis, the glass powder samples were degassed at 250 °C for 6 h in a vacuum process.

#### VSM analysis

Magnetic properties of the Fe-doped MBGs were measured by a vibrating sample magnetometer (VSM) analysis (Lake Shore Cryotronics, USA) using a magnetic field of 20 kOe at room temperature.

#### Electro-Fenton's reaction analysis

##### Preparation of working electrode

**Scheme 1:**
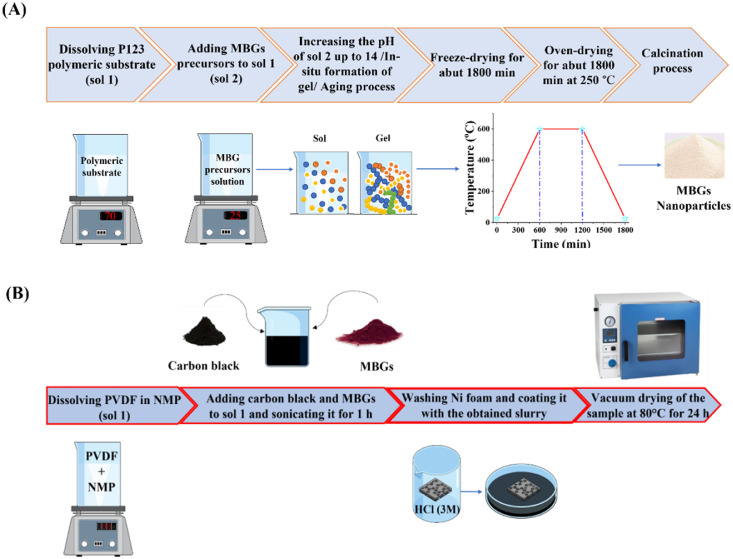
A pictorial illustration of the steps involved in the synthesis process (A) and working electrode preparation process (B).

To prepare working electrode, MBG powders, polyvinylidene fluoride (PVDF, Sigma-Aldrich, USA), and carbon black (Sigma-Aldrich, USA) were mixed in the mass ratio of 75:22:3. To do this, 29.4 mg of polyvinylidene fluoride (PVDF) were dissolved in 1 mL of 1-methyl-2-pyrrolidinone (NMP) under constant stirring for 60 min (sol-A). Then, 100 mg of MBG particles and 4 mg of carbon black were separately added to sol-A every 45 min intervals. The obtained solution was sonicated using a sonication bath for 1 h. Subsequently, the solution was stirred for 24 h in order to reach the appropriate coating slurry. To prepare Ni foam (1 × 0.5 cm^2^), a substrate for coating, first the oxidized layer was removed using HCl solution (3 M), and then it was carefully rinsed with alcohol and deionized water three times. Finally, the dip-coated foam in the slurry was dried using a vacuum process at 80 °C for 24 h. The schematic representation of synthesis steps and working electrode preparation is shown in Scheme 1A and B.

##### Electro-Fenton properties

The electrochemical properties of the prepared working electrode were recorded using an electrochemical analyzer (Autolab PGSTAT302N). In the test, a platinum (Pt) wire, Ag/AgCl, and 1 M Na_2_SO_4_ solution were utilized as the counter electrode, reference electrode, and electrolyte reference for electrochemical measurements at room temperature, respectively. It is worth noting that the electrochemical analysis was recorded in O_2_-saturated electrolyte using a portable oxygen concentrator.

Cyclic voltammetry (CV) analysis of the coated glasses was recorded at a scan rate of 50 mV^.^s^−1^. It should be mentioned that the CV test was analyzed in N_2_-purged and the O_2_-saturated solutions. Electrochemical impedance spectroscopy (EIS) analysis was evaluated using alternating current (AC) at an amplitude of 10 mV in the frequency range of 0.1 Hz–10^5^ Hz. The solution resistance (R_s_), constant phase element (CPE), and charge transfer resistance (R_ct_) of the samples were derived using ZView® software (version 3.5) by fitting the EIS data and simulating impedance measurements.

#### *In vitro* bioactivity assessment

To evaluate the bioactivity, we first prepared the SBF according to Kokubo's method [26]. Then 0.15 g of the synthesized MBGs were incubated in 100 mL of SBF. The solution was shaken at a speed of 20 rpm at the temperature of 37 °C for the periods of 1, 3, and 7 days. Meanwhile, pH values of the glass-containing SBF were recorded by using a digital pH-meter (AZ pH Meter 86,552, Taiwan) over the incubation periods. Besides, the release profile of ions from the glass into SBF was monitored via inductively coupled plasma atomic emission spectroscopy (ICP-AES, Spectro Arcos, Germany).

### Statistical analysis

The particle size and ζ potential results were performed three times, and the obtained data were represented as mean ± standard deviation. The outcomes were statistically analyzed via the one-way ANOVA analysis (GraphPad Prism, 8.4.3(686), USA) followed by post hoc analysis. (*p ≤ 0.05, **p ≤ 0.01, ***p ≤ 0.001, ****p ≤ 0.0001).

## Results

### DTA/TGA analysis

**Fig. 1:**
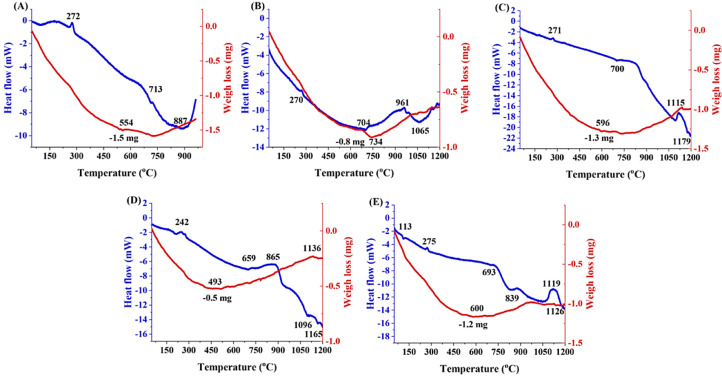
DTA/TGA graphs of Fe_2_O_3_ free (A),  1% (B), 2.5% (C), 5% (D) and 7.5% (E) Fe_2_O_3_-containing MBGs.

Fig. 1 shows the DTA and TGA results of the synthesized glasses after the freeze-drying process. In general, the peaks lower than 300 °C are attributed to physically adsorbed water in the compounds. The minor endothermic peaks around 700 °C could be related to the decomposition of nitrate groups, in good agreement with the results reported by other authors for gel-derived 45S5 glasses [27]. According to DTA data, the glass transition of the Fe-free MBG was around 713 °C while the values recorded for 1, 2.5, 5, and 7.5 mol% Fe_2_O_3_-containing MBGs were about 704, 700, 659, and 693 °C, respectively. It seems that the major weight loss observed in the samples is associated with the burning out of the Pluronic P123 template. Finally, increasing the weight in the TGA graphs can be related to the oxidation of the new phases [28].

**Fig. 2:**
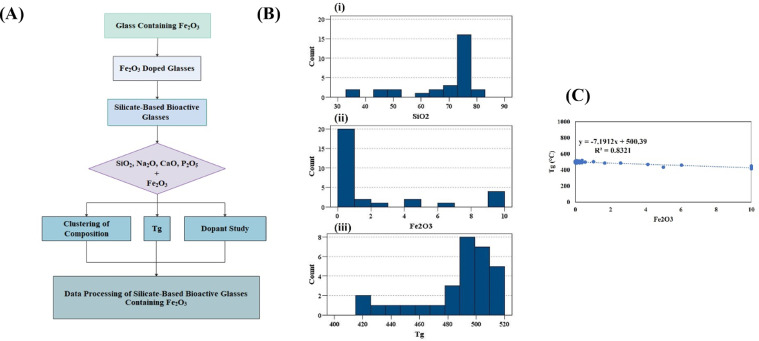
(A) The decision tree of the Fe_2_O_3_-containing silicate glasses (A). The frequency distribution histogram of SiO_2_, Fe_2_O_3_, and Tg (B). The dependency of the Tg values on the Fe_2_O_3_ amounts in the MBGs (C).

Fig. 2A illustrates how we used the Sci-glass data for comparing the synthesized glasses’ Tg with those reported results on Fe_2_O_3_-containing 45S5 composition. The frequency distribution histogram of SiO_2_, Fe_2_O_3_, and T_g_ in the extracted data is shown in Fig. 2B. According to the data, the Tg values have changed after incorporation of Fe_2_O_3_ into MBGs; it shows a decrease along with an increase in Fe_2_O_3_ concentrations (up to 10 mol%), and then it increased again Fig. 2C clarifies that the Tg values (y) are function of the Fe_2_O_3_ concentrations (x) as *y* = 1.11618 x^2^- 22.826 *x* + 528.65.

### DLS and zeta potential characterization

The DLS characterization of the glass particles is represented in Table 2. The Dv 90 value measured for the Fe-free MBG was 110 ± 9 nm, while the values of 86 ± 8, 39 ± 6, 12 ± 1, and 11 ± 1 nm were recorded for 1, 2.5, 5, and 7.5 mol% Fe_2_O_3_-containing samples, respectively. According to the data, the particle size significantly decreased after the incorporation of Fe_2_O_3_ into the glass structure. The ζ potential of the Fe-free sample was about -27 ± 4 mV, while the values of -27 ± 5, -28 ± 2, -30 ± 4, and -27 ± 3 mV were recorded for 1,2.5, 5, and 7 mol% Fe_2_O_3_-doped glasses.

**Table 2:** Particle size and zeta potential values of the Fe free and Fe-doped 45S5 MBGs. (*p ≤ 0.05, **p ≤ 0.01, ***p ≤ 0.001, ****p ≤ 0.0001).

Sample	Dv 50 (nm)	P value	Dv 90 (nm)	P value	Zeta (mV)	P value
Fe0	83 ± 4	–	110 ± 9	–	−27 ± 4	–
Fe1	59 ± 3	**	86 ± 8	**	−27 ± 5	*P* > 0.05
Fe2.5	20 ± 2	***	39 ± 6	***	−28 ± 2	*P* > 0.05
Fe5	6 ± 2	***	12 ± 1	***	−30 ± 4	*P* > 0.05
Fe7.5	6 ± 1	*****	11 ± 1	****	−27 ± 3	*P* > 0.05

### XRD patterns

**Fig. 3:**
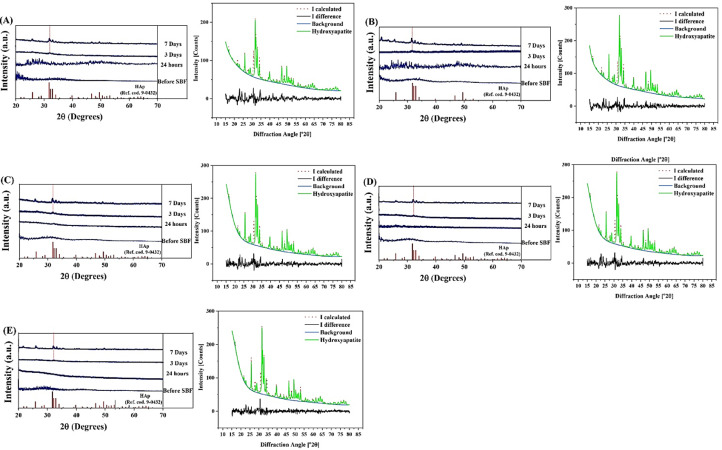
The XRD patterns of Fe_2_O_3_ free (A),  1% (B), 2.5% (C), 5% (D) and 7.5% (E) Fe_2_O_3_-containing MBGsbefore and after immersion in the SBF. The Rietveld refinement results of the immersed samples after 7 days are illustrated in the figures.

The results of the XRD analysis are shown in Fig. 3 before and after immersion in SBF. The figure shows the Rietveld analysis results of the SBF-immersed glasses after 7 days of incubation. Besides, the calculation of crystallinity, crystallite size, lattice constant, and rate of the glass phase transformation to hydroxyapatite (HAp) (i.e., the bioactive properties) are represented in Table 3. According to the obtained data, all the glass samples were amorphous before incubation in SBF. The crystallinity values calculated for the Fe-free, 1, 2.5, 5, and 7.5 mol% Fe_2_O_3_-containing MBGs were 6, 6, 3, 3, and 2%, respectively. The HAp phase (ICCDD ref. cod. 9–0432) was detected in all SBF-immersed samples as 27, 30, 24, 20, and 15%, respectively. According to the data, the lattice constant of the formed HAp is higher in the samples incubated for 7 days as compared to their counterparts after 3 days. The rate constant of glass to HAp transformation in the SBF were 6, 7, 9, 13, and 15 × 10^−7^ s^−1^ for the Fe-free, 1, 2.5, 5, and 7.5 mol% Fe-doped MBGs, respectively.

**Table 3:** Summarized results of crystallite size, crystallinity, lattice constants of the formed HAp, and the average rate constant of glass to HAp transformation.

Sample	Crystallite size (nm)		Crystallinity (%)				a Å		c Å		Average rate constant (K × 10^−7^) S^−1^
Days	3	7	0	1	3	7	3	7	3	7	–
F0	8	48	6	5	8	27	9.418	9.422	6.881	6.883	6
F1	9	60	6	5	10	30	9.429	9.431	6.890	6.890	7
F2.5	5	51.4	3	3	6	24	9.432	9.434	6.895	6.898	9
F5	6	52.4	3	3	5	20	9.436	9.443	6.900	6.904	13
F7.5	6	49.6	4	2	4	15	9.430	9.432	6.893	6.893	15

### FTIR spectroscopy

**Fig. 4:**
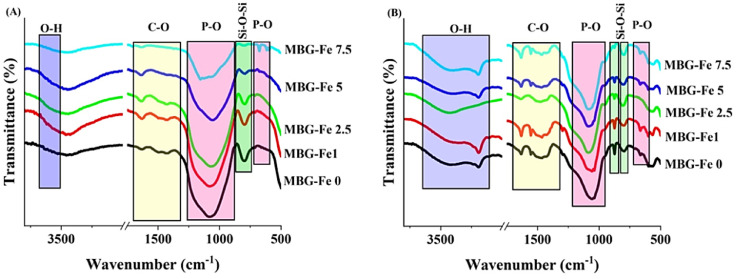
FTIR spectra of the undoped and Fe_2_O_3_-doped MBGs before (A) and after 7 days of incubation in SBF (B).

Fig. 4 displays the FTIR spectra of the synthesized MBGs before and after immersion in SBF. The marked bands in the range of 553–680 cm^−1^ and the broadband around 1100 cm^−1^ are related to P-O bonds [29]. The bands around 800 cm^−1^ and 870 cm^−1^ are associated with structural groups of the silicate glass [28]. As it can be seen, the intensity of the Si-O-Si groups shows a reduction in the Fe-doped samples due to changes that occurred in the glass network. The observed broad bands in the range of 1460–1560 cm^−1^ are attributed to carbonated groups. The increased intensity of the carbonated groups in the SBF-immersed MBGs is correlated to the formation of the hydroxycarbonate apatite (HCA) layer.

### FE-SEM observations

**Fig. 5:**
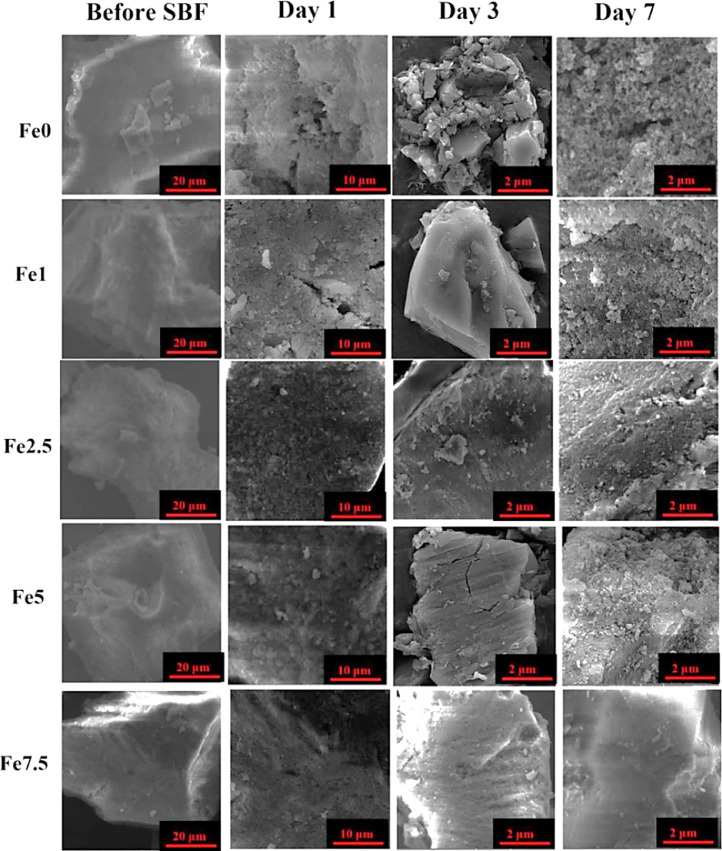
SEM micrographs of the undoped and Fe_2_O_3_-doped MBGs before and after incubation in the SBF for time periods up to 7 days.

The surface morphology of the MBGs is presented in Fig. 5. The formation of typical HCA agglomerates onto the SBF-immersed samples can be clearly observed in the relative images, indicating the bioactive feature of all the samples.

### TEM images

**Fig. 6:**
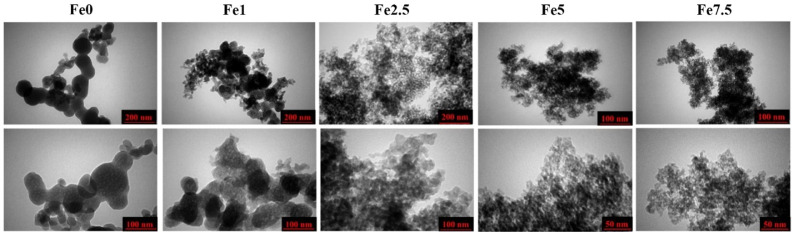
TEM micrographs of the Fe_2_O_3_ free and Fe_2_O_3_-doped MBGs.

TEM micrographs of the un-doped and Fe-doped MBGs are shown in Fig. 6. A porous structure was observed in all the glass particles. As can be observed in the TEM images, the particle size of the Fe-doped samples was reduced, and its agglomeration was increased.

### AFM micrographs

**Fig. 7:**
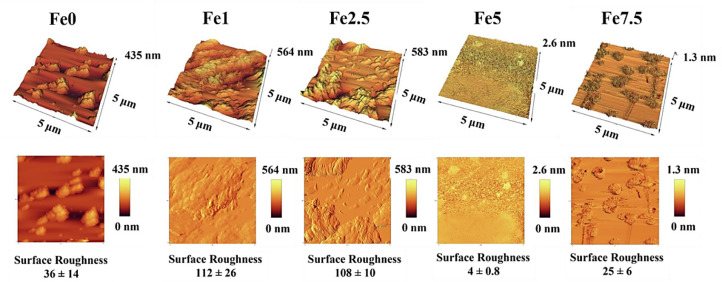
AFM micrographs of the Fe_2_O_3_ free and Fe_2_O_3_-containing MBGs.

The surface roughness of the glass particles (Fig. 7) was monitored by using AFM microscopy. As shown, the addition of Fe_2_O_3_ to the parent 45S5 glass made significant changes in the surface roughness of the synthesized MBGs. The average value of surface roughness was 36 ± 14 nm for the Fe-free MBGs, while the values decreased to 112 ± 26, 108 ± 10, 4 ± 0.8, and 25 ± 6 nm for 1, 2.5, 5, and 7.5 mol% Fe_2_O_3_-containing samples, respectively.

### N_2_ adsorption/desorption analysis

**Fig. 8:**
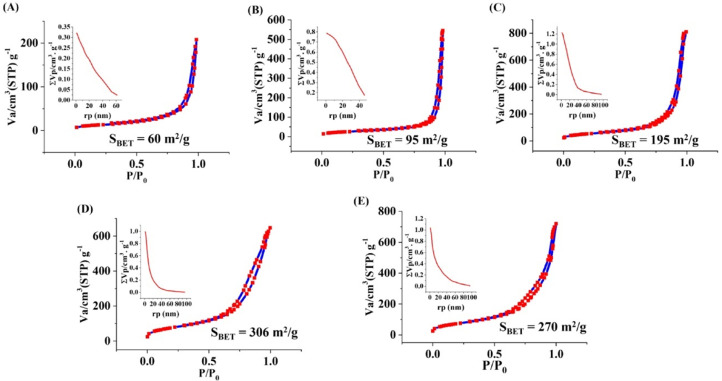
N_2_ adsorption-desorption isotherms, specific surface area by BET and pore size distributions by BJH method of 0% (A), 1% (B), 2.5% (C), 5% (D) and 7.5% (E) Fe_2_O_3_ doped MBGs.

The BET/BJH results of the prepared samples are shown in Fig. 8. According to Brunauer–Deming–Deming–Teller theory (BDDT) classification, the shape of the hysteresis loop of the MBGs is related to category IV and closely attributed to the H4 class of mesoporous particles. This class designates particles with hollow spheres of irregular and broad size and/or ordered mesoporous particles [30]. The details of mesoporous characteristics of the MBGs are presented in Table 4, indicating the mesoporous range (2–50 nm) of materials.

**Table 4:** The mesoporous characteristics of the synthesized Fe_2_O_3_ free and Fe_2_O_3_ doped MBGs.

Sample	S_BET_ (m^2^/g)	Total pore volume (cm^3^/g)
Fe0	60	0.32
Fe1	95	0.84
Fe2.5	195	1.22
Fe5	306	0.99
Fe7.5	270	1.00

### VSM

**Fig. 9:**
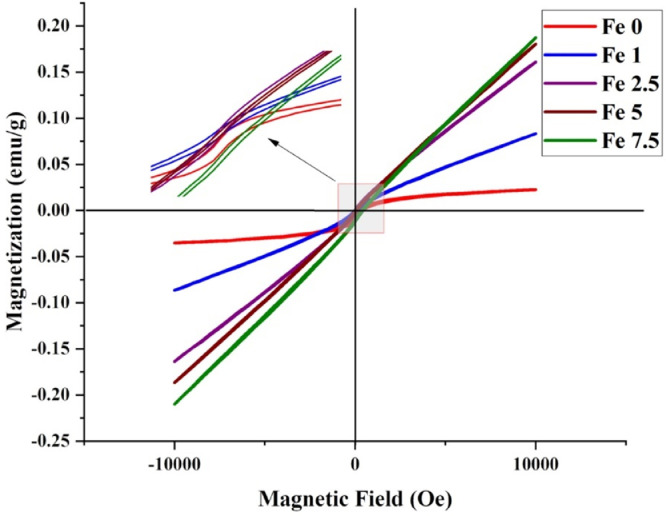
Magnetic hysteresis loops of the Fe_2_O_3_ free and Fe_2_O_3_-containing MBGs.

The magnetic hysteresis (M-H) loops of the MBGs are presented in Fig. 9. The values of magnetization saturation (M_s_) of the samples were 0.021, 0.083, 0.161, 0.180, and 0.187 emu/g for the Fe-free, 1, 2.5, 5, and 7.5 mol% Fe_2_O_3_-containing MBGs, respectively.

### Ion release and pH variations

**Fig. 10:**
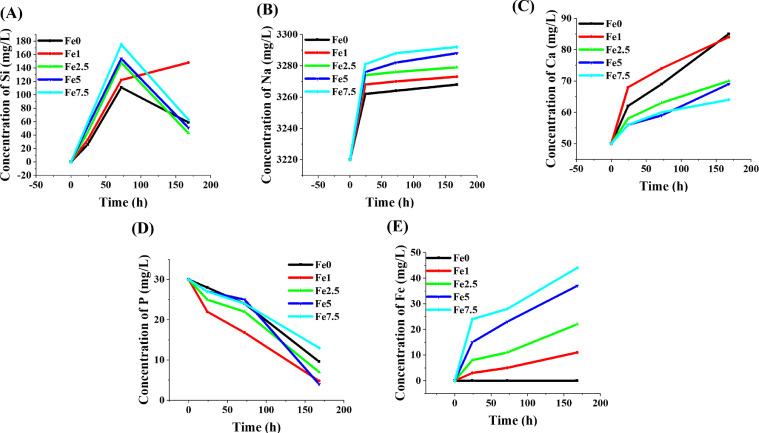
The release of various ions from the Fe_2_O_3_ free and Fe_2_O_3_-containing MBGs into the SBF release medium during 7 days of incubation.

**Fig. 11:**
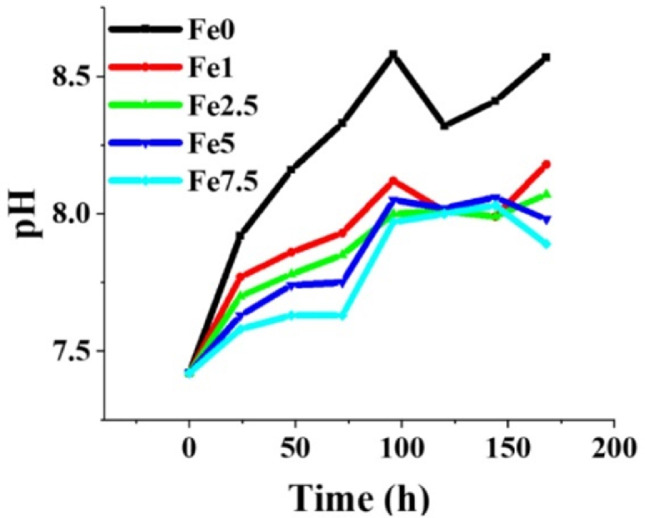
The pH variations during the incubation time of Fe_2_O_3_ free and Fe_2_O_3_-containing MBGs in SBF.

The release profile of ions from the MBGs into SBF is illustrated in Fig. 10. The calculated kinetics of the ions released estimated from the slope of the curves are represented in Supplementary Tables S1–S5. According to the data, the highest release rate values of Fe^3+^ ions are related to the first 24 h of incubation. The pH changes in the glass-containing SBF were recorded for 7 days. As shown in Fig. 11, a sharp increase in the pH (from 7.42 to 8.5) is seen during the first 100 h of post-incubation.

### Electro-Fenton properties

#### Cyclic voltammetry (CV) analysis

**Fig. 12:**
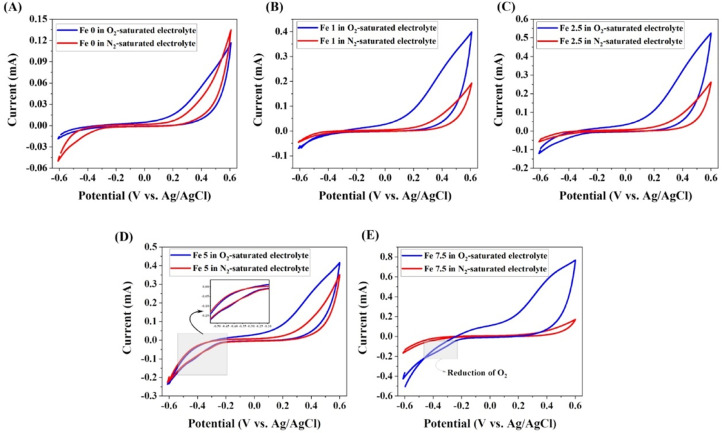
Cyclic voltammetry analyses of the Fe_2_O_3_ free (A), 1% (B), 2.5% (C), 5% (D) and 7.5% (E) Fe_2_O_3_-containing MBGs.

The CV scan of the coated MBGs in both O_2_-and N_2_-saturated electrolytes is represented in Fig. 12. As shown, the reduction peak assigned as reduction of O_2_ and subsequent generation of H_2_O_2_ is only observed in the CV curves of the MBGs incorporating 5 and 7.5 mol% of Fe_2_O_3_ at a cathodic potential higher than −0.2 V (vs. Ag/AgCl) in O_2_-saturated solution. It is clearly visible that the reduction peak is wholly disappeared in N_2_ gas purging conditions.

#### Electrochemical impedance spectroscopy (EIS)

**Fig. 13:**
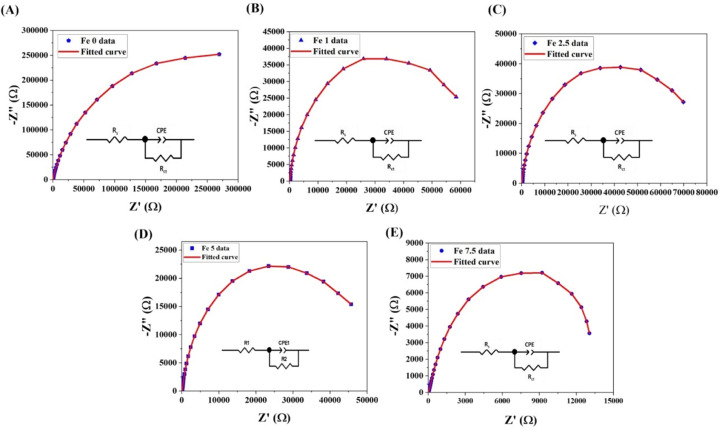
Nyquist plots and EIS equivalent circuit fitting of the Fe_2_O_3_ free (A), 1% (B), 2.5% (C), 5% (D) and 7.5% (E) Fe_2_O_3_-containing MBGs.

Fig. 13 illustrates the EIS profile of the working electrode coated with Fe_2_O_3_-doped MBGs in O_2_-saturated electrolytes. For all the samples, one semicircle is observed in Nyquist plots. This semicircle could be attributed to different kinds of pore geometry. An equivalent circuit, including the electrolyte resistance (R_s_), charge transfer resistance (R_ct_), and constant phase element (CPE) is seen in Figs. 12 and 13. The R_s_, R_ct_, and CPE values of five different working electrodes from Nyquist plots are summarized in Table 5. Moreover, the rate constant (K^0^) values of the samples are represented in Table 5. The K^0^ of electrodes coated with the MBGs were 6.25 × 10^−10^, 4.47 × 10^−9^, 4.55 × 10^−9^, 7.57 × 10^−9^, 2.40492 × 10^−8^ for the un-doped, 1, 2.5, 5, and 7.5 mol% Fe_2_O_3_-containing samples, respectively.

**Table 5:** The EIS equivalent circuit fitting data (R_s_, CPE, and R_ct_) and calculated rate constants of electro-Fenton reaction (K^0^).

Sample	R_s_ (Ohm)	CPE ( × 10^−5^)	R_ct_ (Ohm)	K^0^
Fe0	65.62	1.73	7.09 × 10^5^	6.25 × 10^−10^
Fe1	55.55	6.57	9.92 × 10^4^	4.47 × 10^−9^
Fe2.5	62.19	5.17	9.74 × 10^4^	4.55 × 10^−9^
Fe5	66.58	8.06	5.86 × 10^4^	7.57 × 10^−9^
Fe7.5	26.36	18.17	1.84 × 10^4^	2.40 × 10^−8^

## Discussion

In recent years, glasses and glass-ceramics have attracted much attention in cancer treatment due to their great functional versatility and outstanding features, including excellent biocompatibility, the ability to load anticancer drugs and chemicals, and the possibility of producing radioactive glasses [11,12]. Furthermore, adding anticancer elements to the glass structure is a fairly simple and feasible approach with satisfactory outcomes in battling against cancer cells. Fe and iron oxides are well-known anticancer substances, which can generate ROS in tumor cells through the reduction of H_2_O_2_ to free radicals (^•^OH) [31]. In the current study, we synthesized a group of sol-gel-derived MBGs based on 45S5 Bioglass formulation in which various concentrations (0, 1, 2.5, 5, and 7.5 mol%) of Fe_2_O_3_ were incorporated in order to obtain anticancer Fe_2_O_3_-containing glasses. As reported elsewhere [32], the P-O-Fe and Si-O-Fe linkages could be formed in the glass structure by adding Fe_2_O_3_ as a modifier oxide. Fe_2_O_3_ (Fe/O = 1.5) can trigger the reduction reactions **(**R1-R8**)** reported below. Regarding the reactions, the standard electrochemical reduction potential of Fe_2_O_3_ (hematite) is higher than other Fe-based oxides including Fe_3_O_4_ (magnetite) and FeO (wüstite).

$$Fe_{2}O_{3}=2Fe^{2+}+4e^{-}+1.5O_{2}\left(g\right)\;E\;\text{vs}\;\text{SHE}=-1.511\;V\;R1$$

$$Fe_{2}O_{3}=2Fe^{3+}+6e^{-}+1.5O_{2}\left(g\right)\;E\;\text{vs}\;\text{SHE}=-1.223\;V\;R2$$

$$Fe_{3}O_{4}=3Fe^{2+}+6e^{-}+2O_{2}\left(g\right)\;E\;\text{vs}\;\text{SHE}=-1.336\;V\;R3$$

$$Fe_{3}O_{4}=3Fe^{3+}+9e^{-}+2O_{2}\left(g\right)\;E\;\text{vs}\;\text{SHE}=-1.011\;V\;R4$$

$$\text{FeO}=Fe^{2+}+2e^{-}+0.5O_{2}\left(g\right)\;E\;\text{vs}\;\text{SHE}=-0.855\;V\;R5$$

$$\text{FeO}=Fe^{3+}+3e^{-}+0.5O_{2}\left(g\right)\;E\;\text{vs}\;\text{SHE}=-0.786\;V\;R6$$

$$\text{Fe}=Fe^{3+}+3e^{-}\;E\;\text{vs}\;\text{SHE}=0.055\;V\;R7$$

$$\text{Fe}=Fe^{2+}+2e^{-}\;E\;\text{vs}\;\text{SHE}=0.407\;V\;R8$$

The DTA analysis (Fig. 1) indicated that the incorporation of Fe_2_O_3_ to the MBGs network can reduce the glass transition temperature (from 713 to 659 °C), which can be associated with an increasing number of non-bridging oxygens (NBOs) in the glass network and subsequent decrease of crystallization energy [33]. The different peak positions seen in DTA graphs could also be related to the anti-polarization effect of Fe^3+^ ions and the decrease of glass network coherency [32]. The particle size analysis (Table 2) indicates that the addition of Fe_2_O_3_ significantly hinders the particle growth (size decrease from 110 to 11 nm), which is in line with other reported experiments on different glass compositions [28]. It should be mentioned that the net negative surface charge of the particles (Table 2) (about −30 mV) could be beneficial for enhancing the biological performance of the MBGs [29].

The XRD results (Fig. 3) suggested that the crystallization rates of the synthesized MBGs was depending on the Fe_2_O_3_ concentration. The rate constant of phase transformation increased along with higher concentrations of Fe_2_O_3_, which may be attributed to the weakening of the structural bonds as a result of the effect of adding a transition metal [34]. Compared to the 3-day SBF-incubated glasses, the Rietveld refinement data showed an increase in the lattice constant of HAp (*a* = b from 9.418 to 9.443 Å, c from 6.881 to 6.904 Å) of the 7-day SBF-immersed samples during the phase transformation of the glass to HAp. This increase could be related to the elevated levels of released ions (Si^4+^, Na^+^, and Fe^3+^) from the MBG particles at day 7 and entrance to the HAp structure [30]. The FTIR results (Fig. 4A) revealed that the intensity and peak positions of the P-O and Si-O-Si bands changed depending on Fe_2_O_3_ content. The formation of P-O-Fe and Si-O-Fe bonds, which occurs randomly, may lead to the disordering of glass structure (higher than its Fe-free pristine form) as well as enhanced cross-link density of the glass [35], resulting in the observed changes in the position and the intensity of FTIR bands. The structural bonds of the HAp/HCA were also observed in Fig. 4B, indicating the bioactivity of the synthesized MBGs.

The SEM micrographs (Fig. 5) also verified the formation of the HCA layer onto the SBF-incubated MBGs after 7 days. The TEM micrographs (Fig. 6) revealed that the presence of Fe_2_O_3_ may decrease the particles size of the MBGs from 50 to 100 nm to below 10 nm. This decrease, also observed in the DLS data, could be attributed to the Fe_2_O_3_ impact on increasing the repulsion force between the glass particles [36]. The AFM data (Fig. 7) revealed that the dopant would enhance the surface roughness from 36 to 112 nm [37]. Therefore, this suggests that the dopant amount in the glass matrix can also have an effect on surface topography and, hence, play a role in modulating the initial interaction of the glass in the biological solution or bio-fluids. The data of the N_2_ adsorption/desorption analysis (Fig. 8) revealed the well-ordered porous structure of the MBGs. This state can be related to the templating action and subsequent burning out of Pluronic P123 during the heat-treatment process. The BET data indicated a significant increase in S_BET_ of Fe-doped samples (from 60 to 306 m^2^/g), which is in line with previous studies [20] and also makes Fe_2_O_3_-containing MBGs suitable for drug delivery applications [38]. The synthesized MBGs possess a certain magnetization saturation (Fig. 9), ranging from 0.021 to 0.187 emu/g, which might be probably attributed to the formation of the ferrite phase (XFe_2_O_4_) due to the transformation of glass to spinel structure [39]. The formation of spinel structure (AB_2_O_4_) in which A site occupied with divalent cations and B occupied with trivalent cations in the random forms could enhance the magnetic properties [40]. However, these phases did not observe in the XRD patterns due to the instrumental detection limitations.

This micromagnetic behavior in all Fe-doped MBGs, which becomes more evident as the F_2_O_3_ content increases, reflects the presence of long-range (Ferri)magnetic interactions among the Fe ions that have been incorporated in the glass network [41]. In fact, two types of interactions exist in glassy systems containing Fe_2_O_3_ as network modifiers, i.e., dipole-dipole and superexchange-type interactions [42]. Overall, the values of magnetization saturation assessed in the present work (<0.2 emu/g) are comparable with those reported for different Fe-doped MBG systems (Fe_2_O_3_ < 10 mol%) by other authors [43,44].

The ICP results (Fig. 10) showed that incorporation of Fe_2_O_3_ had no adverse effects on the release profile of ions from the glass into the surrounding medium (SBF). Apparently, an increasing Fe_2_O_3_ content overall induces less significant increments of pH in the solution. Sequestration of phosphate ions, which is typically related to the formation of a HAp layer on the biomaterial surface, can be seen for all compositions (Fig. 10D), in perfect agreement with the results of *in vitro* bioactivity tests. In addition, a sustained release was monitored for Fe in case of the Fe_2_O_3_-containing MBGs over 7 days, which is in favor of cancer treatment strategies. The indirect electro-oxidation method, i.e., the EF approach, was considered to investigate the *in-situ* production of H_2_O_2_ and Fe^2+^ in a two-electron oxygen reduction reaction (ORR). For evaluating the EF activity of Fe_2_O_3_-containing 45S5 MBGs, we employed the CV and the EIS analyses in the 0.1 M O_2_- and N_2_-saturated Na_2_SO_4_ solution. In the mentioned tests, the reduction (cathodic) peak is regarded as one of the most important indicators of EF activity in the coated samples. In this study, the cathodic peak is attributed to the reduction of O_2_ into H_2_O_2_ (R9):

$$O_{2}+2H^{+}+2e^{-}\to H_{2}O_{2}\;\left(R9\right)$$

The Fe^X+^ ions released from the MBGs (Fig. 10F) and the Fenton's reaction occurred as follows (R10):

$$Fe^{2+}+H_{2}O_{2}\to Fe^{3+}+OH^{-}{+}^{•}\text{OH}\;\left(R10\right)$$

In Fenton's reaction, free radicals (^•^OH) are generated at sufficient levels for inducing cancer cell death. It is noted that the formation of free radicals is also feasible in another approach such as Haber–Weiss reaction, as follows (R11):

$${}^{•}{O^{2}}^{-}+H_{2}O_{2}\to OH^{-}{+}^{•}\text{OH}+O_{2}\;\left(R11\right)$$

In addition, according to the following reaction (R12), the released Fe^X+^ ions from the MBGs can be regenerated continuously during the process of the EF reaction. Potential changes during this reaction also may be a critical factor in killing cancer cells.

$$Fe^{3+}+e^{-}\to Fe^{2+}\;E\;\text{vs}\;\text{SHE}=0.649\;V\;\left(R12\right)$$

We further used the EIS test for determining the kinetics of the EF activities in Fe-doped MBG powders. The EIS is a powerful method for the assessment of EF kinetics in different electrodes [45]. Moreover, the various equivalent circuits were used for the physical evaluation of diverse reactions in the EF reaction as well as the clarification of electrolyte-electrode interference. In the Nyquist plots (Fig. 13), one semicircle is observed in all the samples. This semicircle could be attributed to the presence of different kinds of pore geometry in the samples, which was also observed in BET data. It is claimed that the observation of only one semicircle in the EIS curve may be associated with the open superficial morphology that inhibits the pore contribution on the impedance spectra [46].

Another criterion for evaluating the EF reaction is its standard rate constant (K^0^), which is calculated from the following equation (Eq. (2)):

(2) $$K^{0}=\frac{RT}{n^{2}F^{2}R_{ct}AC_{O_{2}}}$$

Where R is the gas ideal constant (8.314 J/mol^.^K), T is the absolute temperature (298 K), n is the mol number during two-electron reduction of O_2_, F is the Faraday's constant (96,485 C/mol), A is the area of the working electrode (0.5 cm^2^), and $C_{O_{2}}$ is the bulk concentration of dissolved oxygen (3 × 10^−7^ mol/cm^3^).

The obtained data based on Eq. (2) are summarized in Table 5. According to the information, the EF kinetics of the MBGs containing 7.5 mol% Fe_2_O_3_ were 3.18, 5.28, 5.38, and 38.44 times higher than the doped samples with 5, 2.5, 1, and 0 mol% Fe_2_O_3_, respectively. Regarding the EIS data, which are in good agreement with the CV results, the EF properties of the MBGs are enhanced by increasing the dopant amount. As a result, it can be concluded that the MBGs incorporating 7.5 mol% of Fe_2_O_3_ have superior EF activities compared to other electrodes, proofing a concept for further *in vitro* and *in vivo* studies.

## Conclusions

A series of Fe-doped MBGs were successfully synthesized based on 45S5 bioglass formulation through the sol-gel process in the presence of the Pluronic P123 template for potential use in cancer treatment. We supposed that these compositions can activate the Fenton's reaction in cancer cells through generating free radicals (^•^OH). The obtained results indicated that adding Fe_2_O_3_ to the glass may result in a slight decrease in bioactivity *in vitro*. The synthesized Fe-doped MBGs showed a particle size between 11 and 86 nm, surface charge value of 27–30 mV, S_BET_ of 95–306 m^2^/g, and Ms of 0.08 to 0.2 emu/g. The Fe_2_O_3_ incorporation into the glass network could alter the kinetics of phase transformation of glass to HAp as well as the ion release kinetics. The electrochemical investigation, including the CV and the EIS, confirmed the high potential of the Fe-doped MBGs in activating the Fenton's reaction. Starting from these highly-promising obtained results, further *in vivo* animal studies seem essential in order to conclude the actual potential of Fe-doped MBGs in cancer therapy strategies.

## Funding

This study was kindly funded by Mashhad University of Medical Sciences through research grant number 4000105.

## CRediT authorship contribution statement

**Farzad Kermani:** Investigation, Writing – original draft. **Arghavan Vojdani-Saghir:** Investigation, Writing – original draft. **Sahar Mollazadeh Beidokhti:** Validation, Writing – review & editing. **Simin Nazarnezhad:** Investigation, Writing – original draft. **Zahra Mollaei:** Formal analysis. **Sepideh Hamzehlou:** Writing – review & editing. **Ahmed El-Fiqi:** Conceptualization, Validation, Writing – review & editing. **Francesco Baino:** Validation, Writing – review & editing. **Saeid Kargozar:** Funding acquisition, Project administration, Resources, Supervision, Writing – review & editing.

## Declaration of Competing Interest

The authors have no conflict to declare.

## Appendix Supplementary materials

Image, application 1
