# Supplementary material for: Iron (Fe)-doped mesoporous 45S5 bioactive glasses: Implications for cancer therapy
Source: Transl Oncol. 2022 Mar 30;20:101397. doi: 10.1016/j.tranon.2022.101397 (PMC8972012; doi:10.1016/j.tranon.2022.101397)
Supplement: Supplementary file 1 [file mmc1.pdf]

Table S1: The calculated kinetic of Si-ions releasing during 168 h of incubations.

	Si				
**Time**	Fe 0	Fe 1	Fe 2.5	Fe 5	Fe 7.5
**0-24**	1.08	1.375	1.83	2.20	2.43
**24-72**	1.77	1.85	2.16	2.10	2.45
**72-168**	-0.54	0.27	-1.10	-1.07	-1.16
**Average**	0.77	1.16	0.97	1.08	1.23
**Increase compared to MBG-Fe 0**	0	51.35	25.70	40.10	60.36

Table S2: The calculated kinetic of Na-ions releasing during 168 h of incubations.

	Na				
**Time**	Fe 0	Fe 1	Fe 2.5	Fe 5	Fe 7.5
**0-24**	1.75	2	2.25	2.33	2.54
**24-72**	0.042	0.042	0.041	0.13	0.14
**72-168**	0.042	0.031	0.031	0.06	0.041
**Average**	0.61	0.69	0.77	0.84	0.91
**Increase compared to MBG-Fe 1**	0	13.06	26.70	37.5	48.86

Table S3: The calculated kinetic of Ca-ions releasing during 168 h of incubations.

	Ca				
**Time**	Fe 0	Fe 1	Fe 2.5	Fe 5	Fe 7.5
**0-24**	0.5	0.75	0.34	0.25	0.25
**24-72**	0.14	0.12	0.10	0.06	0.09
**72-168**	0.16	0.10	0.07	0.10	0.04
**Average**	0.27	0.33	0.17	0.13	0.12
**Increase compared to MBG-Fe 0**	0	20.51	-37.17	-48.71	-53.84

Table S4: The calculated kinetic of P-ions releasing during 168 h of incubations.

	**P**				
**Time**	Fe 0	Fe 1	Fe 2.5	Fe 5	Fe 7.5
**0-24**	-0.09	-0.33	-0.21	-0.21	-0.13
**24-72**	-0.08	-0.11	-0.10	-0.04	-0.10
**72-168**	-0.15	-0.13	-0.16	-0.13	-0.11
**Average**	-0.10	-0.18	-0.15	-0.13	-0.10
**Increase compared to MBG-Fe 0**	0	78.95	34.90	21.71	-4.60

Table S5: The calculated kinetic of Fe-ions releasing during 168 h of incubations.

	**Fe**				
**Time**	Fe 0	Fe 1	Fe 2.5	Fe 5	Fe 7.5
**0-24**	0	0.12	0.33	0.62	1
**24-72**	0	0.04	0.06	0.16	0.08
**72-168**	0	0.06	0.11	0.15	0.16
**Average**	0	0.07	0.17	0.32	0.42
**Increase compared to MBG-Fe 1**	-	0	122.72	309.10	445.41
